# Heart rate variability and cognitive functions in adolescents with complex congenital heart disease

**DOI:** 10.1038/s41390-024-03432-9

**Published:** 2024-07-30

**Authors:** Asuka Toyofuku, Melanie Ehrler, Nadja Naef, Alenka S. Schmid, Oliver Kretschmar, Beatrice Latal, Ruth O’Gorman Tuura

**Affiliations:** 1 Child Development Center, University Children’s Hospital Zurich, Zürich, Switzerland; 2 Children’s Research Centre, University Children’s Hospital Zurich, Zürich, Switzerland; 3 Centre for MR Research, University Children’s Hospital Zurich, Zürich, Switzerland; 4 Department of Surgery, Pediatric Cardiology, Pediatric Heart Center, University Children’s Hospital Zurich, Zürich, Switzerland; 5 University Research Priority Program (URPP), Adaptive Brain Circuits in Development and Learning (AdaBD), University of Zurich, Zürich, Switzerland

## Abstract

**Background:**

Heart Rate Variability (HRV) originates from the interplay between parasympathetic/sympathetic inputs to the heart, thus serving as an indicator of Autonomic Nervous System regulation. Prior research indicates that decreased HRV, marked by reduced autonomic balance, is related to poorer cognitive performance. While the population with congenital heart disease (CHD) show changes in HRV linked with the heart defect, the association between HRV and cognitive functions in CHD remains unexplored.

**Methods:**

46 adolescents with CHD who went through infant open-heart surgery and 64 healthy controls (50.9% males, 12.8 ± 1.4 years) underwent neurodevelopmental testing and photoplethysmograph acquisition. Group differences and associations with cognitive functions were analysed with linear regression. *P* values were FDR-corrected.

**Results:**

Adolescents with CHD showed lower HRV (quantified by high-frequency power) compared to controls (*p* < 0.001). Lower HRV was correlated with worse executive function (*β* = 0.24, *p* = 0.044) and lower IQ (*β* = 0.26, *p* = 0.010) in the whole sample and with lower IQ (*β* = 0.35, *p* = 0.014) in the CHD group. These associations were robust to confounders, including age, sex, and socioeconomic status.

**Conclusion:**

Our findings demonstrate an association between HRV and cognitive functions in adolescents with complex CHD. Early detection of alterations in HRV/autonomic regulation may help to identify children with CHD at risk for cognitive impairments.

**Impact:**

- Adolescents with congenital heart disease (CHD) showed lower heart rate variability (HRV), indicating an imbalanced autonomic nervous system.
- Lower HRV was associated with lower IQ and executive function (EF) in the whole sample.
- The association between HRV and IQ was significantly stronger in CHD than in healthy controls.
- This study provides the first evidence of a link between altered HRV and cognitive impairments in the CHD population.
- Neurodevelopmental impairments seen in adolescents with CHD could be linked to their altered cardiac autonomic nervous activity, marked by low HRV.

## Introduction

The autonomic nervous system (ANS) regulates the body’s internal environment and maintains physiological homoeostasis. It operates involuntarily, governing bodily functions with two components: the sympathetic and parasympathetic branches. The sympathetic nervous system (SNS) is responsible for the “fight or flight” response and increases heart rate, cardiac muscle contractility, blood pressure, and breathing frequency. The parasympathetic nervous system (PNS), on the other hand, is marked by the “rest and digest” response and has the opposite effects to those of the SNS.^1^

One marker for ANS activity is heart rate variability (HRV), which quantifies the variation in time intervals between consecutive heartbeats (R-R intervals).^2^ HRV originates from the sympathetic and parasympathetic inputs to the heart through the sinoatrial node. This variability in the heart rate reflects the status of the ANS, which enables the heart and the body to adjust rapidly to psychological and environmental changes.^3^ The status of ANS and cardiac conditions are closely connected. Altered HRV is associated with a variety of cardiovascular diseases and is typically characterised by elevated sympathetic activity and hypoactive parasympathetic activity.^4^

HRV is measured in the time domain and frequency domain. Time domain indices quantify the amount of variability observed during monitoring periods ranging from 1 min to 24 h. The major time domain measurements include RMSSD, NN50, and pNN50. RMSSD (ms) represents the root mean square of successive differences between normal heartbeats.^2^ NN50 is defined by the number of consecutive heartbeat intervals that differ by more than 50 ms, and pNN50(%) represents the percentage of NN50 during the recorded time period. RMSSD and pNN50 are suggested to reflect the vagus nerve activity (vagal tone), which is the main nerve of the PNS.^5,6^ In the frequency domain, the signal is filtered into distinct frequency bands: Low-Frequency (LF) and High-Frequency (HF) rhythms. LF rhythms (0.04–0.15 Hz) are modulated by sympathetic activity, while HF rhythms (0.15–0.4 Hz) are modulated by parasympathetic activity,^7^ although LF is not exclusively sympathetic activity driven.^8^

Importantly, HRV is not a static measure but exhibits significant variation due to multiple factors. HRV generally increases from infancy until adolescence.^9,10^ After reaching adulthood, HRV gradually decreases with age, reflecting the natural decline in parasympathetic tone and an increase in sympathetic dominance.^11^ The same age-related changes in HRV can also be observed in patients with CHD.^12^ Furthermore, sex might affect HRV in healthy children and adults.^13,14^ HRV is also influenced by respiration and blood pressure sensors. While inhaling, the heart rate increases, and blood pressure decreases seconds later, and while exhaling, the heart rate drops and blood pressure increases seconds later, too. This rhythmic fluctuation in heart rate synchronised to inspiration and expiration is called Respiratory sinus arrhythmia (RSA).^15^ RSA is suggested to reflect the status of the parasympathetic activities or vagal tone together with HF power.

Interestingly, HRV has recently garnered substantial attention in association with cognitive function. A growing body of research suggests that HRV is not merely an indicator of autonomic balance but also a correlate of cognitive performance.^16^ Studies have revealed a consistent positive association between higher HRV and better cognitive functions, spanning domains such as executive function, memory function, and attention. This HRV-cognition association was found not only in healthy populations.^17^ but also in those with neurodegenerative disease.^18^ and populations with cardiac risk factors.^19^ This might indicate that the PFC and subcortical regions, such as the amygdala and brainstem, play a key role in regulating both cognitive functioning and autonomic balance.^4,20^

Congenital Heart Disease (CHD) encompasses a range of structural defects in the heart chambers or the major blood vessels near the heart. It is the most prevalent type of birth defect, occurring at a rate of 8–10 cases per 1000 live births.^21^ Not surprisingly, individuals with CHD are reported to show altered HRV, which could be part of the pathophysiology, compensation mechanism or results of surgery.^22^ Numerous studies have documented reduced HRV in individuals with CHD across different age groups, from fetuses and infants to adults.^23–26^ It has also been reported that individuals with more severe CHD demonstrated lower HRV compared to the simple CHD group,^27^ indicating that the severity of heart defect could impact the cardiac nervous system and the regulation of ANS.

Individuals with CHD are at risk for a spectrum of neurodevelopmental impairments during their school years,^28,29^ and these difficulties may persist into adulthood.^30,31^ Moreover, previous research has demonstrated that populations with CHD might suffer from brain injury, such as white matter injury or stroke, during the neonatal period.^32,33^ Individuals with CHD tend to exhibit lower total and regional brain volume and alterations in the white matter microstructure and structural networks. These structural alterations or brain injury are associated with lower IQ, memory and executive performance.^34,35^ The mechanisms for these alterations are multifactorial and related to clinical risk factors (e.g., number of surgeries, length of hospital stay) and altered intrauterine brain perfusion, leading to poorer brain growth and a higher vulnerability towards neonatal and perioperative hemodynamic changes.^36^

While alterations in HRV have been reported for CHD patients, the association between HRV and cognitive functions in the CHD population is unknown. The purpose of this study is to evaluate the link between HRV and cognitive functions in the adolescent CHD population.

## Method and materials

### Participants

This analysis constitutes a component of a prospective cohort study.^37^ The primary focus of this study is to examine the link between cognitive impairments and brain development in adolescents with CHD. The data collection was completed at the University Children’s Hospital of Zurich from April 2019 to September 2021.

Patients with a complex CHD who underwent cardiopulmonary bypass surgery (CPB) between 2004 and 2012 at the University Children’s Hospital Zurich were eligible for inclusion in the study if: (1) they underwent CPB surgery before one year of age, (2) do not have a genetic or dysmorphic syndrome, and (3) they were between 10 and 15 years of age at the time of examination. In total, 100 adolescents with CHD partook in the current study (participation rate: 56%) out of 178 eligible patients. As a control group, 104 healthy adolescents between 10 and 15 years of age were also recruited. The exclusion criteria for the control group were: (1) born before 36 weeks of gestation, (2) diagnosis of a neurological or substantial developmental disorder (i.e., learning disorder or attention deficit hyperactivity disorder). Of the 204 participants recruited into the study, 156 (*N* = 60 CHD patients and *N* = 96 controls) underwent MRI. The recruitment process is documented in an earlier study,^38^ and a flow chart describing the excluded cases is included in the Supplementary Fig. 1.

The approval of the study was granted by the ethics committee of the Canton of Zurich. Prior to participation, explicit written consent was acquired from the participant’s legal guardian, as well as from participants aged 14 years and above.

### Photoplethysmography data acquisition and HRV analysis

#### Photoplethysmography (PPG) data acquisition

PPG is a widely used non-invasive technique that utilises infrared light to measure the volumetric variations of blood circulation, heart rate, and pulse rate. During a resting state fMRI sequence, the heart rate and respiratory rate were monitored with PPG and a breathing belt, respectively. The duration of the resting fMRI was 6 min 19 s, and the PPG recording was 6–7 min, beginning during the pre-scans and ending with completion of the scan. Each participant had one recording except three participants who had two recordings due to motion or the scan having been stopped early. In case more than one recording was available, the recording with fewer artefacts were chosen. A PPG sensor was placed on the participants’ index finger, and throughout the scan, they remained lying down with their eyes closed. Participants were mostly relaxed and acclimatised to the scanner during the recording since the resting state scan was performed after a series of structural scans. All participants were provided with an emergency buzzer, so that they could alert the study team in case they felt stressed or anxious.

#### HRV data processing

The data from the PPG sensor were extracted from the scanner for offline processing. In order to compute the HRV, the PPG data was analysed with the fully automated KUBIOS HRV PREMIUM 3.5.0 software (Biosignal Analysis and Medical Imaging Group, Department of Applied Physics, University of Eastern Finland, Kuopio, Finland.^39^). R-R intervals were registered over a period of 5 min for analysis: the heart rate was calculated as the average heart rate during the five-minute measurement (short-term HRV recordings).

Peaks in the PPG data, detected by the Kubios software with a beat-detection algorithm,^40^ were visually checked by the authors for quality control purposes. Any subjects with more than ten missing beats and subjects with a data duration of less than 4 min were omitted. We used the automatic beat correction algorithm, which corrects ectopic or misplaced beats and thus improves the accuracy of HRV calculation.^40^

Among the calculated HRV parameters, RMSSD (ms) and pNN50 (%) from the time domain and High-Frequency power (log) and Low-Frequency power (log) from the frequency domain were included. In the frequency domain, the HRV spectrum is calculated with Fast Fourier Transform based on Welch’s periodogram method and then log-transformed in the software. RSA was derived from PPG data and Respiratory data using Matlab R 2022a (9.12.0.2009381). RSA was computed by the peak-trough method, where RSA is defined as the difference between the shortest inter-beat interval (IBI) during heart rate acceleration in the inspiration phase and the longest IBI during deceleration in the expiration phase.^41^

### Neuropsychological assessment

#### Intelligence Quotient (IQ)

To assess IQ, we employed the short version of the Wechsler Intelligence Scale for Children 4th edition (WISC-IV) in German.^42^ The WISC-IV short form was corrected according to an equation.^43^ and demonstrated a strong correlation with the full version (*r* > 0.90). The assessment comprised a combination of the subsets Matrices, Similarities, Letter Number Sequencing, and Symbol Search subtests.

#### Executive Functions (EF)

EF in various domains, including working memory, inhibition, cognitive flexibility, planning, and fluency, were assessed with a comprehensive neuropsychological test battery.^37,38^ The Delis-Kaplan Executive Function System,^44^ the Test of Attentional Performance (TAP),^45^ and the Regensburger Verbal Fluency Test.^46^ were utilised. Details about the neuropsychological test measures of EFs are displayed in Table 1. A summary score for overall EF performance was calculated using the control population as the norm. The tests were completed in a randomised order to mitigate the influence of fatigue and motivation loss. Trained psychologists and paediatricians from the Child Development Center at the University Children’s Hospital Zurich conducted and interpreted the tests.

**Table 1:** Neuropsychological test battery to assess executive functions performance.

Domains	Neuropsychological test	Test measurement
Working memory	Digit span forward and backward (WISC-IV)	No. of correct items
	Letter number sequencing (WISC-IV)	No. of correct items
	Corsi block tapping test (Corsi)	No. of correct items
Inhibition	Inhibition Subtest interference, colour word interference task (D-KEFS)	Completion time
	Go/NoGo (TAP)	No. of commission errors
Cognitive flexibility	Subtest letter-number-switching, trail making task (D-KEFS)	Completion time
	TAP flexibility (TAP)	Median reaction time
Fluency	Subtests s-words and animals (RWT)	No. of correct items
	Subtest filled-dots-only, design fluency test (D-KEFS)	No. of correct items
Planning	Tower task (D-KEFS)	Total achievement score

#### Other assessments

Socioeconomic status (SES) was quantified by parental education. Parental education was measured with a 6-point Likert scale, which was used from 1 = no high school qualification to 6 = university degree. SES was calculated by the sum of the highest paternal and maternal education, ranging from 2 to 12.

Clinical risk factors were collected from the patient’s clinical records, such as the length of intensive care unit (ICU) stay, the number of open-heart surgeries, having been on ECMO, having had a stroke, having had a seizure, and the use of cardiac medication at the time of assessment. We used the length of ICU stay as a proxy for clinical risk factors, as factors like the number of surgeries or the severity of the disease are associated with the length of ICU stay.

### Statistical analysis

All statistical analyses were performed using R software (version 3.4.2). Initially, the demographic characteristics of the CHD group and the control group were examined. Independent *t* tests were utilised to analyse variables such as age, IQ, EF summary score, and EF domains. Mann–Whitney U test was used to analyse SES distribution. The distribution of males and females within the groups was examined using a chi-squared test. There were missing cases in SES (12/100 in the CHD group and 12/104 in the control group), and median imputation was employed for each group to maintain the sample size. The subset of individuals who did not have HRV data or were excluded due to artefacts was compared to the whole sample to examine if there was any bias in population characteristics (age, sex, SES, and IQ).

Next, the interrelationships of HRV parameters (RMSSD, pNN50, HF power, LF power, and RSA) were investigated by calculating the correlation matrix. The presence of outliers was assessed by plotting HRV parameters against age. The data distribution of four HRV parameters and RSA were examined with density plots. For those variables with non-normal distributions, the normality of the residuals was analysed visually using QQ plots to determine whether parametric tests were appropriate.

All the statistical analyses were conducted while controlling for covariates, including age, sex, and SES. Group differences in HRV parameters between the CHD and the control group were evaluated using multiple linear regression. Additionally, group differences among different CHD subgroups (univentricular or biventricular; cyanotic or acyanotic) in HRV parameters were explored using a similar approach. Group differences by severity according to the criteria of Warnes et al. (simple, moderate, or severe).^47^ were analysed using one-way Analysis of Variance (ANOVA). To investigate if there is any effect of clinical risk factors on HRV indices in the CHD group, the association between HRV indices and the length of ICU stay was examined. Interaction effects of group, age, sex, and SES on HRV indices were analysed through a multiple linear regression model to examine if the relationship between the group and the HRV indices varies depending on the levels of other variables such as age, sex, and SES. Effects of the length of ICU stay, in addition to the above-mentioned variables, were analysed in the CHD group.

HRV parameters that significantly differed between patients and controls were associated with cognitive outcomes (IQ and EF summary score) and were estimated using multiple linear regression. Cognitive outcomes were set as dependent variables, while HRV parameters were considered independent variables. Additionally, the associations between individual EF domain scores and HRV indices were investigated in a similar way. *P* values were false discovery rate (FDR) corrected. Interaction effects were tested to investigate if the strength of association between HRV and cognitive outcomes differs between patients and controls. If the interaction terms were statistically significant, a subgroup analysis was conducted with multiple linear regression. A subgroup analysis for the CHD group was conducted while adjusting for the clinical risk factors (i.e., the length of ICU stay). Statistical significance was determined at a two-tailed *p* < 0.05.

#### The use of Large Language Models (LLMs)

Chat GPT 3.5 was used to check the R code and correct the grammar and vocabulary.

## Results

### Demographics

The final sample size consisted of 110 participants (46 CHD patients and 64 healthy controls) after excluding 94 participants (CHD = 54, control = 40) due to artefacts or lack of HRV data (Supplementary Fig. 1). The mean age of the final sample was 12.80 years (standard deviation (SD): 1.38). There was no difference in sex distribution between groups. The CHD group had lower SES than the control group (CHD median: 8.0, control median: 9.5, *p* < 0.001). Compared to controls, patients with CHD scored significantly lower in most neurodevelopmental tests. The mean scores (with SDs) of each variable are shown in Table 2. The clinical characteristics of patients and CHD diagnosis are shown in Table 3 and Supplementary Table 1. The subset of individuals excluded due to artefacts or lack of HRV data (*N* = 94) had a mean age of 12.83 years (SD = 1.36). This excluded subset was not significantly different from the final sample used in this study in terms of age, sex and SES. However, this excluded subset showed statistically lower IQ (mean = 102.80, SD = 14.64) compared to the final sample (mean = 107.22, SD = 11.82) (*p* = 0.024) and CHD subjects in the excluded subset spent longer days in ICU (mean = 17.15, SD = 31.81) compared to the ones in the final sample (mean = 7.37, SD = 3.59) (*p* = 0.029).

**Table 2:** Demographic data of participants.

Variables	CHD (*n* = 46)	Controls (*n* = 64)	*p* value
Age, mean (SD)	13.22 (1.05)	12.50 (1.51)	0.004
Male sex, No.(%)	27 (58.70)	29 (45.31)	0.233^a^
SES, median (IQR)	8 (6–9)	9.5 (8-11)	<0.001^b^
Gestational age, mean (SD), wk	39.46 (1.69)	39.32 (1.43)	0.692
IQ, mean (SD)	100.91 (12.34)	111.90 (8.97)	<0.001
EF summary score, mean (SD), z	−0.89 (1.13)	0.02 (0.93)	<0.001
Working memory, mean (SD), z	−0.97 (1.30)	0.16 (0.85)	<0.001
Inhibition, mean (SD), z	−0.54 (1.05)	−0.01 (1.14)	0.013
Flexibility, mean (SD), z	−0.98 (1.23)	−0.01 (1.01)	<0.001
Fluency, mean (SD), z	−0.38 (1.29)	−0.05 (0.99)	0.150
Planning, mean (SD), z	−0.24 (1.09)	−0.02 (1.03)	0.301

Others: Two-sampled independent t-test.

^a^Two-sampled χ^2^ test.

^b^Two-sampled Mann–Whitney U test.

**Table 3:** Characteristics of CHD patients.

Variable	Characteristics
Prenatal diagnosis, No. (%)	10 (21.7)
Cyanotic/Acyanotic	
Cyanotic, No. (%)	31 (67.4)
Acyanotic, No. (%)	15 (32.6)
Type of ventricle	
univentricular, No. (%)	8 (17.4)
biventricular, No. (%)	38 (82.6)
Severity^a^	
Simple, No. (%)	9 (19.6)
Moderate, No. (%)	12 (26.1)
Severe, No. (%)	25 (54.3)
Perioperative time (first surgery)	
Preoperative saturation, mean (SD)	89.5 (13.2)
Age at surgery, mean (SD), month	2.7 (3.1)
Lowest temperature, mean (SD), °C	28.9 (3.9)
Time on ECC, mean (SD), min	160.1 (71.9)
Hospitalization days, median (IQR)	21.5 (15.6–33.0)
Days in ICU, median (IQR)	7.00 (5–9)
No. CPB surgical procedures, median (IQR)	1.00 (1–1.8)
Critical neurological or cardiac event (first surgery)	
Postoperative ECMO, No. (%)	2 (4.3)
Clinically symptomatic seizure, No. (%)	3 (6.5)
Stroke identified on cerebral MRI, No. (%)	6 (13.0)
Cardiac medication at follow-up assessment No. (%)	27 (58.7)

*CHD* congenital heart disease, *CBP* cardiopulmonary bypass, *ECC* extracorporeal circulation, *ICU* intensive care unit.

^a^Severity: severity of the disease categorised by Warnes.^47^

### HRV

The data distribution of four HRV parameters and RSA were examined. Except for LF power, the rest of the variables were not normally distributed; thus, the residuals’ normality was visually analysed using QQ plots. All of the variables’ residuals turned out to be normally distributed; therefore, parametric tests were employed for the rest of the HRV analysis.

**Fig. 1:**
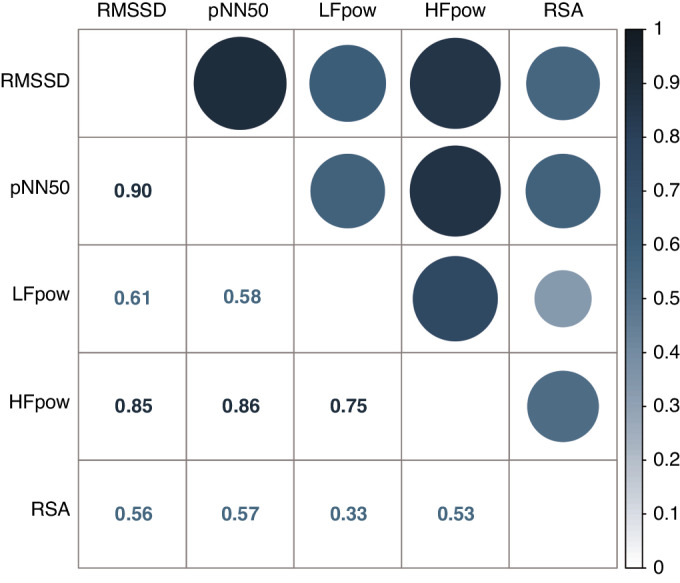
HRV intercorrelation. Pearson correlation was used (Pearson correlation coefficient (r) is shown). LFpow LF power, HFpow HF power. RMSSD and pNN50 are time-domain indices, while HF/LF power are frequency-domain indices.

Next, the interrelationships of HRV parameters were presented in a correlation matrix (Fig. 1). The HRV parameters within the same domain (time domain: RMSSD and pNN50, frequency domain: HF power and LF power) were highly interrelated as expected. While HF power is highly interrelated to the time domain HRV indices, LF power is less strongly correlated. Furthermore, RSA was weakly correlated to the HRV parameters, especially to the LF power.

**Fig. 2:**
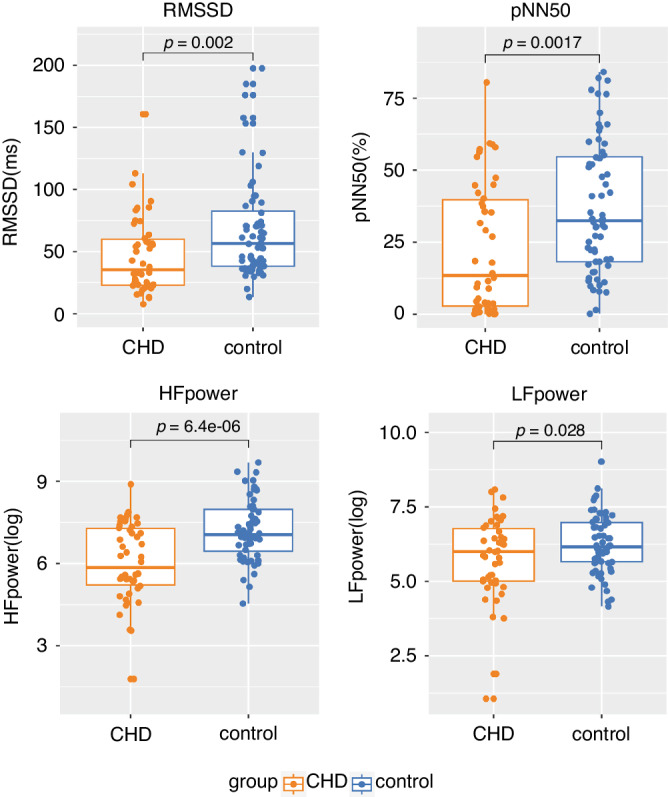
Group differences in HRV indices. The CHD group had consistently lower values across the four HRV indices than the control group. Orange dots = CHD group, Blue dots = control group. Covariates (age, sex and SES) are controlled. P-values before FDR correction are shown here. All the HRV indices survived FDR correction.

Patients with CHD showed statistically lower values in RMSSD, pNN50, HF power and LF power compared to controls (Fig. 2). There were no significant group differences in RSA. (See Table 4). Furthermore, interaction effects of group, age, sex and SES on HRV indices did not attain statistical significance, indicating that the relationship between HRV indices and the group were not statistically different depending on the level of other covariates (i.e., age, sex and SES).

**Table 4:** Group differences in HRV indices.

	Effect	B	Std. Error	95_CI		*β*	uncorr-p	FDR-corr p
				LL	UL			
RMSSD	(Intercept)	−0.695	38.410				0.986	0.986
	group	24.788	7.860	0.162	0.479	0.321	0.002**	0.004**
	age	3.843	2.641	−0.015	0.291	0.138	0.149	0.258
	sex	−12.229	7.099	−0.309	−0.011	−0.160	0.088	0.220
	SES	0.237	1.710	−0.147	0.175	0.014	0.890	0.901
pNN50	(Intercept)	−11.746	23.349				0.616	0.770
	group	15.411	4.778	0.167	0.479	0.323	0.002**	0.004**
	age	2.300	1.606	−0.017	0.285	0.134	0.155	0.258
	sex	−8.050	4.315	−0.318	−0.024	−0.171	0.065	0.220
	SES	0.969	1.040	−0.067	0.249	0.091	0.354	0.901
HF power	(Intercept)	4.546	1.288				0.001**	0.002**
	group	1.253	0.264	0.315	0.600	0.458	**0.000**^**§**^	**0.000**^**§**^
	age	0.110	0.089	−0.034	0.257	0.112	0.218	0.273
	sex	−0.294	0.238	−0.251	0.033	−0.109	0.219	0.274
	SES	0.020	0.057	−0.120	0.185	0.033	0.728	0.901
LF power	(Intercept)	5.230	1.236				**0.000**^**§**^	**0.000**^**§**^
	group	0.562	0.253	0.067	0.399	0.233	0.028*	0.036*
	age	0.054	0.085	−0.097	0.221	0.062	0.528	0.528
	sex	−0.286	0.228	−0.275	0.034	−0.120	0.213	0.274
	SES	−0.007	0.055	−0.179	0.153	−0.013	0.901	0.901
RSA	(Intercept)	0.271	0.125				0.032*	0.053
	group	0.026	0.025	−0.065	0.277	0.106	0.319	0.319
	age	0.016	0.009	0.022	0.339	0.180	0.072	0.258
	sex	−0.004	0.023	−0.174	0.141	−0.017	0.865	0.865
	SES	−0.004	0.006	−0.236	0.098	−0.069	0.506	0.901

^*^p < 0.05, **p < 0.01, ^§^*p* < 0.0001.

Values that meet *p* < 0.05 (considered significant in this study) are marked in bold.

**Fig. 3:**
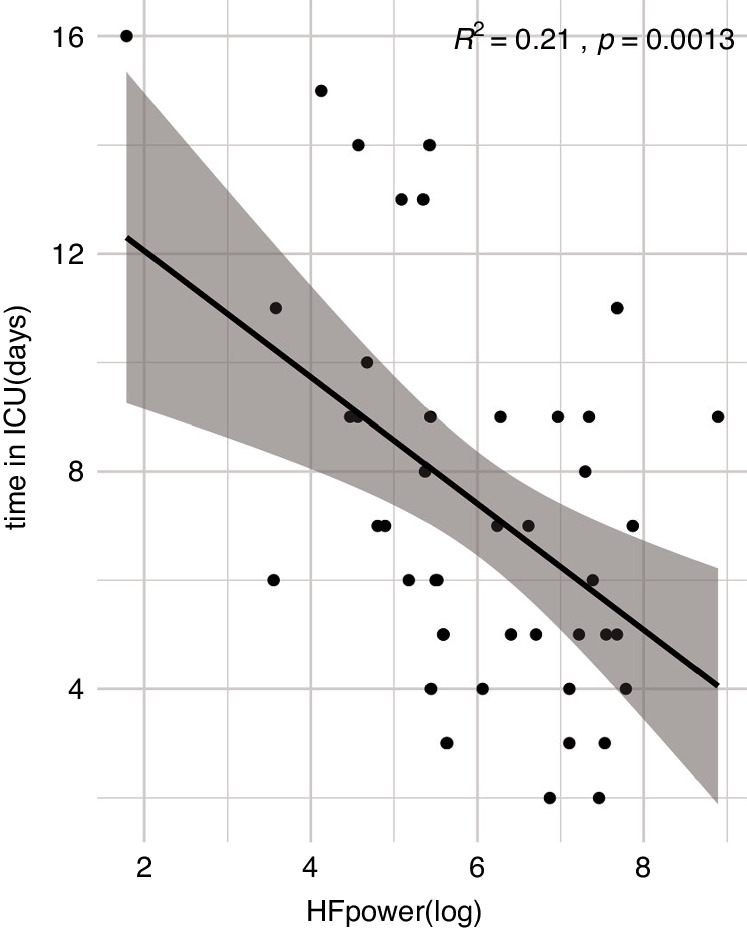
Linear regression of HF power and the length of ICU stay. The length of ICU stay is negatively associated with HF power in the CHD sample (*N* = 46). ICU intensive care unit.

Across all HRV parameters, no group differences were found between the univentricular and the biventricular CHD. Adolescents with cyanotic CHD had slightly lower HF power than acyanotic CHD (*β* = 0.33, 95_CI[0.084, 0.574], *p* = 0.048), but there were no group differences in other HRV indices. With ANOVA, no group differences were found among CHD groups categorised by their severity (simple, moderate and severe), according to Warnes.^47^ However, the length of ICU stay during infancy, which reflects the severity of the disease, was associated with HRV indices; RMSSD (*β* = −0.329, 95_CI[−0.55,1 −0.107], *p* = 0.035), pNN50 (*β* = −0.398, 95_CI[−0.608, −0.187], *p* = 0.014), HF power (*β* = −0.474, 95_CI[−0.665, −0.283], *p* = 0.005), LF power (*β* = −0.320, 95_CI[−0.541, −0.099], *p* = 0.035), RSA (*β* = −0.407, 95_CI[−0.617, −0.198], *p* = 0.014). HF power was especially strongly correlated with the length of ICU stay (Fig. 3).

### HRV and cognitive functions

**Fig. 4:**
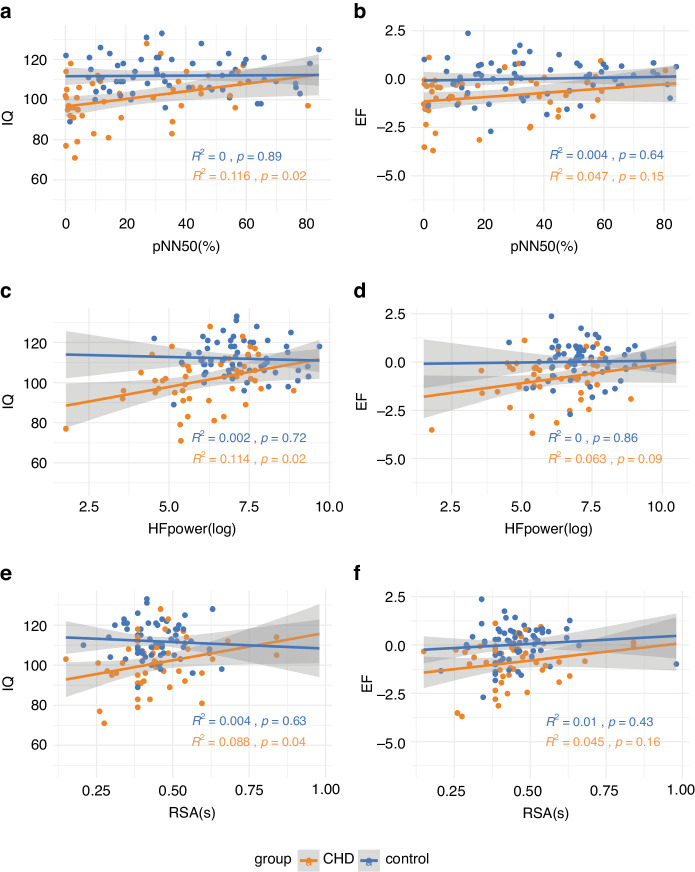
linear regression of HRV indices and cognitive functions for CHD and controls. **a** pNN50~IQ, **b**: pNN50~EF, **c**: HF power~IQ, **d**: HF power~EF, **e**: RSA ~ IQ, **f**: RSA ~ EF. Orange dots = CHD group, Blue dots = control group. The CHD group consistently showed a positive association between HRV indices (pNN50, HFpower and RSA) and cognitive functions (IQ or EF). In contrast, the control group showed no statistically significant correlation even though positive associations were found in the whole sample (*N* = 110). The unit of EF is expressed with a z-score.

Over the whole sample, pNN50 was significantly associated with IQ (*p* = 0.023), HF power with IQ (*p* = 0.010) and EF summary score (*p* = 0.044), and RSA with IQ (*p* = 0.023). See Tables 5 and 6. Figure 4 reports these associations separately for CHD and controls). The association between RMSSD and IQ (*β* = 0.167, uncorrected *p* = 0.049), pNN50 and EF summary score (*β* = 0.186, uncorrected *p* = 0.043), RSA and EF summary score (*β* = 0.197, uncorrected *p* = 0.029) did not survive the FDR correction. RMSSD and EF were not correlated. LF power was not correlated with either IQ or EF.

**Table 5:** Multiple regression for IQ and HRV indices across the whole sample (*N* = 110).

	Effect	*B*	Std.error	95_CI		*β*	uncorr p	FDR-corr p	R^2^	p model fit
				LL	UL					
IQ ~ RMSSD	(Intercept)	96.585	10.673				**0.000**	**0.000**^**§**^		
	RMSSD	0.052	0.026	0.033	0.301	0.167	**0.049**	0.061		
	age	−1.138	0.730	−0.264	0.005	−0.130	0.122	0.139	0.270	**0.000**^**§**^
	sex	−0.319	1.975	−0.150	0.123	−0.014	0.872	0.951		
	SES	2.539	0.452	0.353	0.597	0.475	**0.000**	**0.000**^**§**^		
IQ~pNN50	(Intercept)	97.293	10.509				**0.000**	**0.000**^**§**^		
	pNN50	0.110	0.042	0.084	0.350	0.217	**0.011**	0.023*		
	age	−1.151	0.721	−0.264	0.001	−0.131	0.113	0.139	0.288	**0.000**^**§**^
	sex	−0.120	1.953	−0.140	0.129	−0.005	0.951	0.951		
	SES	2.426	0.450	0.330	0.578	0.454	**0.000**	**0.000**^**§**^		
IQ~HFpower	(Intercept)	84.982	11.177				**0.000**	**0.000**^**§**^		
	HFpower	2.245	0.711	0.128	0.387	0.258	**0.002**	0.010*		
	age	−1.068	0.710	−0.253	0.009	−0.122	0.136	0.139	0.308	**0.000**^**§**^
	sex	−0.416	1.913	−0.149	0.114	-0.018	0.828	0.951		
	SES	2.397	0.443	0.326	0.571	0.449	**0.000**	**0.000**^**§**^		
IQ ~ RSA	(Intercept)	89.473	11.099				**0.000**	**0.000**^**§**^		
	RSA	22.537	8.994	0.075	0.339	0.207	**0.014**	0.023*		
	age	−1.240	0.725	−0.275	-0.008	−0.142	0.090	0.139	0.285	**0.000**^**§**^
	sex	−0.986	1.943	−0.176	0.092	−0.042	0.613	0.951		
	SES	2.735	0.446	0.393	0.631	0.512	**0.000**	**0.000**^**§**^		

R^2^ Adjusted R-squared. ^*^p < 0.05, ^§^*p* < 0.0001.

Values that meet *p* < 0.05 (considered significant in this study) are marked in bold.

**Table 6:** Multiple regression for EF and HRV indices across the whole sample (*N* = 110).

	Effect	B	Std.error	95_CI		β	uncorr p	FDR-corr p	R^2^	p model fit
				LL	UL					
EF~pNN50	(Intercept)	−1.159	1.051				0.273	0.273		
	pNN50	0.009	0.004	0.041	0.330	0.186	**0.043**	0.072		
	age	−0.076	0.072	−0.239	0.050	−0.095	0.292	0.354	0.154	**0.0002**^**†**^
	sex	0.028	0.199	−0.133	0.158	0.013	0.889	0.982		
	SES	0.168	0.046	0.195	0.476	0.336	**0.000**	**0.000**^**§**^		
EF~HFpower	(Intercept)	−2.234	1.115				**0.048**	0.213		
	HFpower	0.193	0.072	0.096	0.376	0.236	**0.009**	0.044*		
	age	−0.069	0.071	−0.228	0.056	−0.086	0.331	0.354	0.176	**0.000**^**§**^
	sex	0.006	0.195	−0.140	0.146	0.003	0.975	0.982		
	SES	0.164	0.045	0.190	0.468	0.329	**0.000**	**0.000**^**§**^		
EF ~ RSA	(Intercept)	−1.682	1.076				0.121	0.213		
	RSA	1.834	0.830	0.055	0.339	0.197	**0.029**	0.072		
	age	−0.091	0.072	−0.258	0.032	−0.113	0.210	0.354	0.159	**0.0002**^**†**^
	sex	−0.028	0.196	−0.157	0.131	−0.013	0.886	0.982		
	SES	0.189	0.045	0.244	0.514	0.379	**0.000**	**0.000**^**§**^		

^*^p < 0.05, ^§^*p* < 0.0001.

Values that meet *p* < 0.05 (considered significant in this study) are marked in bold.

Interaction effects showed that the association between pNN50 and IQ (*β* = −0.391, 95_CI[−0.672, −0.110], *p* = 0.029), HF power and IQ (*β* = −1.04, 95_CI[−1.785, −0.293], *p* = 0.029) and RSA and IQ (*β* = −0.746, 95_CI[−1.300, −0.192], *p* = 0.034) were stronger in patients compared to controls. The group-specific β coefficients show the difference in the strength and direction of associations (i.e., pNN50 ~ IQ: CHD *β* = 0.361, control *β* = −0.025, HF power ~ IQ: CHD *β* = 0.348, control *β* = −0.062, RSA ~ IQ: CHD *β* = 0.386, control *β* = −0.008). Within the CHD subgroup only, the associations between IQ and pNN50, HF power, and RSA survived FDR correction for multiple comparisons. Unlike for IQ, the associations between EF summary score and pNN50, HF power and RSA were not statistically different in the patient and control groups.

When the length of ICU stay was added as a covariate in the CHD group’s regression model, the associations between IQ and pNN50, HF power, and RSA did not survive FDR correction for multiple comparisons, although effect sizes remained moderate; pNN50~IQ (*β* = 0.314, 95_CI[0.087, 0.541], uncorrected *p* = 0.042), HF power ~ IQ (*β* = 0.303, 95_CI[0.066, 0.540], uncorrected *p* = 0.058), RSA ~ IQ (*β* = 0.343, 95_CI[0.118, 0.569], uncorrected *p* = 0.026).

Interestingly, mediation analysis showed that HF power had a mediation effect on the association between the length of ICU stay and IQ (*β* = −0.134, 95_CI[−0.327, −0.010], *p* = 0.042), and that the direct effect of ICU stay on IQ is not significant without the mediating factor, HF power. The mediation effect of HF power on EF, and pNN50 on IQ/EF were not significant.

To investigate if any of the EF subcategories have driven the summary EF scores, multiple regression was conducted for each EF subcategory. As a whole sample, pNN50 was statistically significantly associated with working memory (*β* = 0.239, 95_CI[0.102,0.377], *p* = 0.017) and planning (*β* = 0.290, 95_CI[0.140,0.440], *p* = 0.016). Inhibition, fluency and cognitive flexibility did not survive the FDR correction. On the other hand, HF power was statistically significantly associated with working memory (*β* = 0.297, 95_CI[0.166,0.429], *p* = 0.003), flexibility (*β* = 0.210, 95_CI[0.067,0.353], *p* = 0.036) and planning (*β* = 0.288, 95_CI[0.140,0.437], *p* = 0.008). Inhibition and fluency did not survive the FDR correction. RSA was not correlated with EF subcategories except for planning (*β* = 0.30, 95_CI[0.153, 0.447], *p* = 0.010).

## Discussion

In the present study, we examined HRV as an indicator of ANS regulation in adolescents with CHD and healthy controls, as well as the associations between HRV and cognitive functions. Our study revealed that adolescents with CHD showed significantly lower HRV (i.e., RMSSD, pNN50, HF and LF power) compared to a healthy control group. Furthermore, HF power was positively associated with IQ and executive functions, and PNN50 was associated with IQ in the whole sample. We identified significant interaction effects, indicating that the association between HRV and IQ was stronger in CHD than in controls. Our findings suggest that increased HRV is associated with better cognitive functions, while lower HRV is associated with lower total IQ, especially among adolescents with CHD.

### HRV in the CHD population

Our findings confirmed that adolescents with CHD showed statistically lower values in all HRV indices, in line with previous reports. Decreased HRV among the CHD population has been commonly reported across different ages, from infants to adults. Most studies reported lower values in both time and frequency domains, and adolescents and adults with CHD are shown to have lower LF/HF ratio,^25^ RMSSD, SDNN, pNN50, LF and HF power.^48^ Our results also confirmed the previous report that HF power is strongly correlated with a time-domain index such as pNN50.^49^

Interestingly, our study did not show statistical differences among CHD severity groups in any HRV parameters. Some previous studies suggest that differences in HRV in CHD subgroups can depend on the severity of the disease. They report lower HRV among severe CHD populations in comparison to groups with simple or moderate CHD.^27,50^ The absence of group differences in our study might be attributed to our relatively small sample size in the CHD severity subgroups (simple; *N* = 9, moderate; *N* = 12, severe; *N* = 25). Furthermore, in the present study, there were no statistically significant differences in HRV indices between the univentricular and the biventricular groups. Adolescents with cyanotic type CHD showed statistically lower HF power than acyanotic type, but there were no group differences in other HRV indices. However, we found statistically significant negative relationships between all HRV indices and the length of ICU stay, which correlates with the severity of the disease. Despite the lack of CHD-type group differences in HRV indices, this indicates that clinical risk factors such as prolonged ICU stay are linked with worse HRV indices and, thus, worse autonomic balance. Studies with a larger sample size among CHD groups would be necessary to examine the association between CHD severity and HRV.

### HRV and cognitive functions

Our study is the first to report the HRV-cognition relationship in patients with CHD. There is a large body of evidence that reduced HRV is associated with poorer cognitive functions in healthy individuals.^17^ and in different clinical cohorts.^18,19^ Our findings offer new insight into previous work, providing the first evidence that these associations are present in adolescents with CHD, as well as over the whole sample population. It is important to note that these associations remained significant even after controlling for the potential confounding factors in our research, as HRV is known to vary by age,^51^ sex.^13,52^ and even SES.^53^

We further looked into the individual executive function domains, such as working memory, flexibility, fluency, inhibition and planning, to clarify if one specific domain of EF drove the significant association between HRV and EF summary score. We found that HRV (e.g., HF power) was associated with working memory, flexibility, and planning in the whole sample. However, no single domain appeared to drive this association, possibly due to the high multicollinearity of EF subdomains and the difficulties experienced in all EF areas by CHD children.^29^ One interesting difference between our results and those reported previously is that in our sample, the inhibition score, one of the components of EF, was not correlated to any of the HRV parameters, while other studies found a connection between HRV and inhibition.^54,55^ In the study by Yang et al., participants with lower HRV showed a lower degree of inhibition when engaging in a highly demanding working memory task.^55^ This difference in results could be attributed to a different measurement of inhibition since inhibition in our study was assessed from the Colour-Word inference and Go/No Go tasks, while in the study by Yang et al., it was assessed by measuring the degree of inhibition of startle response. Thus, we might have captured different aspects of inhibition.

### Difference between CHD and healthy adolescents

We identified significant interaction effects, suggesting the group difference in the strength and direction of the relationships between pNN50/HF power/RSA and IQ. On the other hand, the group interaction effects were not seen in the relationships between HRV indices and EF summary scores. When analysed by subgroups, adolescents with CHD showed stronger associations between HRV and IQ. However, the relationships between HRV and IQ/EF were not evident in the control group alone, possibly because the control group was high-performing in comparison to a normative population, and the variability in cognitive outcomes within the control group was rather small. Thus, it seems like the CHD population drove the positive association between HRV and cognitive functions in the whole sample. This observation is consistent with similar phenomena reported in brain volumetric studies where the association between brain volume and cognitive function was more evident in the CHD group than in the control group.^56,57^

The neurovisceral integration model proposed by Thayer and colleagues offers a valuable framework for understanding the relationship between HRV, ANS and cognitive functions.^5,20^ This model proposes that the interplay between the prefrontal cortex (PFC) and subcortical brain regions (e.g., the amygdala and brainstem) regulate sympathetic and PNS activities, which are reflected in HRV. Furthermore, the PFC plays an important role in higher-order cognitive function; thus, HRV is associated with cognitive processes such as working memory, flexibility, and visuospatial planning.

In CHD patients, the connection between the ANS, HRV and the PFC becomes particularly pertinent due to the impact of the disease/clinical risk factors on ANS regulation. We found that clinical risk factors, such as the length of ICU stay, are negatively associated with HRV indices and that one of the HRV indices (i.e., HF power) mediated the association between the length of ICU stay and IQ. This suggests that part of the adverse effect of prolonged ICU stays on IQ can be explained by alternations in HF power. Moreover, patients with CHD usually go through open heart surgery during infancy, which could induce myocardial damage and impairment of cardiac autonomic nervous activities.^22^ Furthermore, ANS control might have to acclimate to compensate for the altered hemodynamics in the heart and blood vessels. For instance, patients with CHD often display right atrial dilation, elevated right ventricular volume and pressure, low systemic ventricular contractility and a prolonged QRS complex duration.^48,58,59^ These pathophysiologies or surgically induced impairments are connected to the worsening of ANS regulation, elevating sympathetic activities or diminishing parasympathetic activity.

As our study sample demonstrated lower IQ and EF performance among CHD patients, it is often reported that children with CHD encounter a spectrum of neurodevelopmental issues (i.e., lower IQ or lower executive functions).^28,29^ Some of those cognitive issues could be linked to the abnormality in ANS regulation caused by heart defect-related physiology, which impacts the functions in the PFC regions. The PFC alterations in the white matter microstructure.^30^ or white matter injury.^32,60^ were reported to be associated with lower cognitive outcomes among the CHD population.^30,35^ Our study confirmed the connection between lower HRV, lower ANS regulation and poorer cognitive functions in CHD. Future studies should investigate the interplay between PFC alterations, executive function difficulties and disrupted ANS regulation in patients with CHD.

### Limitations and future studies

There are some limitations to this study. Even though our sample size was sufficient to conduct group analyses between CHD and control groups, there were some imbalances in the CHD population regarding disease severity, such that our sample was likely underpowered to detect the effects of disease severity on HRV. The limited sample size and heterogeneity of CHD diagnoses affected the generalisability of this study. Furthermore, elucidating more detailed biological mechanisms underlying HRV-cognition is beyond the scope of this study. Our findings supported the connection between cognitive function difficulties and ANS dysregulation in the CHD population; further studies should investigate the interplay between PFC alterations, executive function difficulties and disrupted ANS regulation in patients with CHD. Ideally, longitudinal designs should be implemented to provide information about causal effects.

## Conclusion

Our study found lower HRV among adolescents with CHD than healthy controls, which was associated with poorer cognitive functions, especially among those with CHD. Populations with CHD have been reported to demonstrate altered cardiac autonomic nervous activity in connection to heart defects, and this study showed the link between dysregulated ANS control marked by low HRV and lower cognitive functions. Our findings provide the first evidence of a link between altered HRV and neurodevelopmental impairments in the CHD population. Future research should elucidate the underlying mechanism of brain-heart bidirectional interactions not only in a healthy population but also among adolescents with CHD who may have altered cardiac ANSs.

## Supplementary information

Supplementary material

## Data Availability

The de-identified data are available from the corresponding author upon reasonable request.
