# Supplementary material for: Heart rate variability and cognitive functions in adolescents with complex congenital heart disease
Source: Pediatr Res. 2024 Jul 30;97(3):1103–13. doi: 10.1038/s41390-024-03432-9 (PMC12055568; doi:10.1038/s41390-024-03432-9)
Supplement: Supplementary file 1 — Supplementary material [file 41390_2024_3432_MOESM1_ESM.pdf]

Supplemental Table 1

Diagnosis	Frequency
ALCAPA (Bland White Garland)	2
Aortic stenosis (valvular)	2
ASD II	1
ASD sinus venosus, ASD II	1
AVSD	1
AVSD complete	3
AVSD unbalanced with right ventricular hypoplasia	1
Coarctation of the aorta (CoA)	2
Crisscross Heart	1
d-TGA	29
d-TGA Subvalvar aortic stenosis	1
DILV	5
DORV	2
DORV Fallot type	1
DORV with TGA	1
DORV with VSD	1
Ebstein's anomaly (severe) + PS	1
Heterotaxy	1
HLHS	8
L-TGA, dextrocardia	1
Pulmonary atresia (PA)	1
PA with intact ventricular septum	1
PA with VSD and aortopulmonary collaterals (MAPCAs)	1
PS valvular	1
Tricuspid atresia (TA)	1
Truncus arteriosus communis (TAC) type 1	4
TAPVC	3
TAPVD	1
Taussig-Bing anomaly	1
Tetralogy of Fallot (TOF)	6
Interrupted aortic arch type B	1
VSD doubly committed	1
VSD malalignment	1
VSD perimembranous	8
VSD perimembranous, ASD II	1
VSD subaortic	1

Abbreviations:
ALCAPA = Anomalous Left Coronary Artery from the Pulmonary Artery, ASD = Atrial Septal Defect, AVSD = Atrioventricular Septal Defect, CoA = Coarctation of the Aorta, d-TGA = Dextro-Transposition of the Great Arteries, DILV = Double Inlet Left Ventricle, DORV = Double Outlet Right Ventricle, HLHS = Hypoplastic Left Heart Syndrome, L-TGA = Levo-Transposition of the Great Arteries, PA = Pulmonary Atresia, PS = Pulmonary Stenosis, TAPVC = Total Anomalous Pulmonary Venous Connection, TAPVD = Total Anomalous Pulmonary Venous Drainage, TA = Tricuspid Atresia, TAC = Truncus Arteriosus Communis, TOF = Tetralogy of Fallot, VSD = Ventricular Septal Defect

Supplemental Figure 1

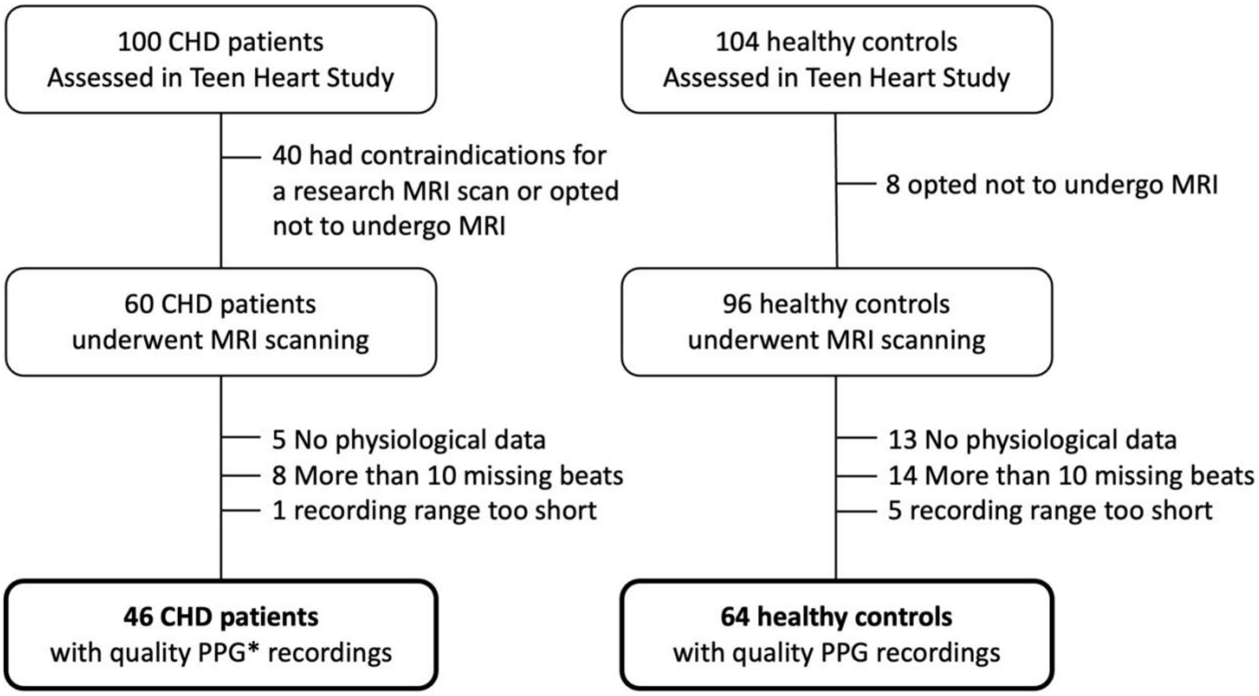

Note: Patients were recruited from longitudinal cohort studies conducted at the University Children's Hospital Zurich. The recruitment process for the Teen Heart Study is documented in von Werdt et al., JAMA Netw Open, 2024. *PPG = Photoplethysmography
